# Medical exercise therapy alone versus arthroscopic partial meniscectomy followed by medical exercise therapy for degenerative meniscal tear: a systematic review and meta-analysis of randomized controlled trials

**DOI:** 10.1186/s13018-020-01741-3

**Published:** 2020-06-15

**Authors:** Jianxiong Ma, Hengting Chen, Aifeng Liu, Yuhong Cui, Xinlong Ma

**Affiliations:** 1 Tianjin Hospital, Tianjin University, Tianjin, 300072 China; 2 Biomechanics Labs of Orthopedics Institute, Tianjin Hospital, Heping District Munan Road NO 155, Tianjin, 300050 China; 3 The First Affiliated Hospital of Tianjin University of Traditional Chinese Medicine, Tianjin, 300110 China; 4 Department of Mechanics, Tianjin University, Tianjin, 300072 China

**Keywords:** Arthroscopic partial meniscectomy, Medical physical therapy, Degenerative meniscal tear, Meta-analysis

## Abstract

**Objective:**

To explore if medical exercise therapy (MET) alone is comparable to arthroscopic partial meniscectomy (APM) followed by MET for knee pain, activity level, and physical function in middle-aged patients with degenerative meniscal tear (DMT) by a systematic review and meta-analysis of randomized controlled trials (RCTs).

**Method:**

A systematic search of electronic databases (PubMed, the Cochrane Library, Embase, and Web of Science) was conducted to retrieve RCTs comparing MET+APM with MET alone for DMT. Risk of bias of the studies was evaluated. Outcomes assessed were pain relief, physical function, and activity level.

**Results:**

A total of 6 RCTs containing 879 patients were included. After pooling the data of 5 researches, we found small significant differences support the APM + MET group for pain control assessed by Knee injury and Osteoarthritis Outcome Score (KOOS) at 2 to 3 months (*p* = 0.004) and at 6 months (*p* = 0.04). And there were statistically improvements in APM + MET at 6 months compared with MET alone when changing measurement to visual analog scale (VAS) (*p* = 0.0003). Our analysis also found small significant differences favor the APM followed by MET group for physical function both at 2 to 3 months (*p* = 0.01, KOOS and Western Ontario and McMaster Universities Osteoarthritis Index, WOMAC; and *P* = 0.40, Lysholm Knee Scoring Scale) and at 6 months (*p* = 0.01, KOOS and WOMAC).

**Conclusion:**

We found favorable results of APM + MET up to 6 months for pain control and physical function. However, there were no differences at longer follow-up. The clinical applicability of APM + MET compared with MET should be interpreted carefully, and the potential of MET to treat DMT should be valued.

## Introduction

The menisci of the knee (medial and lateral) are wedge-shaped semilunar disks which are consisted of fibrocartilage interposed between the condyles of the femur and the tibia [1]. Meniscal tissue is mainly composed of water and type I collagen fibers, which contributes to absorb the energy by converting axial loading forces across the joint into hoop stresses within the tissue. Inevitably, the quality of menisci degenerates with aging: the cellularity, collagen content, and actual amount of glycosaminoglycans diminish, whereas the water content grows [2, 3]. This leads to the meniscus of older individuals that is more prone to acute injuries and chronic damage. Therefore, meniscal tears are badly prevalent, and it was reported that 35% of persons over 50 years old appears imaging evidence of a meniscal tear [4]. Affected individuals clinically present with knee pain, swelling, and impaired function [5].

Great many treatments about degenerative meniscus tears (DMT) have been tried. Currently, arthroscopic partial meniscectomy (APM) has been widely used for patients with DMT [6]. And its popularity has been increasing in many countries [7]. APM therefore contributes significantly to the cost of the healthcare system [8]. In spite of its popularity and the feeling that APM is the standard of care for many meniscal tears, the validity of APM is still controversial. Some non-randomized studies have shown good results in patients with DMT after arthroscopy referring mainly to pain relief, improved knee function, and better quality of life [9, 10]. However, some recent randomized controlled trials (RCTs) of high-quality have showed that APM have rare significant positive effects on patients with meniscal symptoms and knee functions, when it was compared to conventional treatments [11–14] or sham surgery [15, 16]. Recently, medical exercise therapy (MET) has been considered as a prior choice for patients with knee degeneration for purposes of reducing joint pain and improving knee function, both acutely and chronicall y[17, 18]. There are strong evidences that MET has great impacts on relieving symptoms, improving muscle function, and living quality in patients with knee lesions [19], but there is still no consensus about the optimal treatment of DMT up to now. A recent meta-analysis of RCTs found favorable results of APM up to 6 months for physical function and pain relief over a conservative treatment in patients with non-obstructive meniscal tears, but no significant differences at longer follow-up [20]. In order to enhance efficacy, rehabilitation exercises have always been conducted after APM, and these also increase the time and economic cost of patients. However, the efficiency of APM followed by MET compared with MET alone in patients with MET is not yet known.

Therefore, the objective of this study was to decipher the efficacy of MET compared with APM + MET in older patients with non-obstructive meniscal tears.

## Materials and methods

This study was performed according to PRISMA guidelines for systematic reviews and meta-analysis (the PRISMA checklist was provided in Additional file 1) [21].

### Search strategy

Two authors conducted an electronic study screen for RCTs comparing the APM followed by structured exercises with exercises alone in the treatment of degenerative meniscal tear. The electronic databases include PubMed, Embase, web of science, and the Cochrane Library from inception to August 2019. The following terms were used as keywords: exercise, arthroscopic partial meniscectomy, APM, and degenerative meniscal tear (see Additional file 2 for the full search strategy). Besides, further studies were obtained by identifying references of the chosen studies.

### Inclusion criteria

We extracted only clinical RCTs that comparing APM followed by MET with MET alone to treat degenerative meniscal tear. Review articles and case reports were not included and considered for analysis. The outcome variables were knee pain, physical function, activity level, the incidence of complications and adverse events, general health, and living quality. No language and publication restrictions were applied. Eligible studies were evaluated separately by two reviewers. In case of discrepancies, a consensus was reached through discussion.

### Data extraction

A standard data extraction form was employed separately by two authors to collect the information from the studies, including author, study design, publishing date, sample size, follow-up time, patients’, and treatments’ characteristics. Follow-up studies of the same population were regarded as one. For continuous outcomes, we extracted the means with standard deviations. When necessary, we calculated missing standard deviations from other available data according to the formula in Cochrane Handbook for Systematic Reviews of Interventions [22]. The data were collected separately by two authors, and any disagreement was evaluated by the corresponding author.

### Assessment of risk of bias

Seven aspects of the studies related to the risk of bias were evaluated, based on the guidelines in the Cochrane Handbook for Systematic Reviews of Interventions [22]. Two reviewers respectively assessed the risk of bias of all the extracted studies. The assessment was conducted for the domains, including random sequence generation, allocation concealment, blinding of participants and personnel, blinding of outcome assessment, incomplete outcome data, selective outcome reporting, and other bias.

### Statistical analysis

Review Manager Software for Windows (Version 5.3. Copenhagen: The Nordic Cochrane Centre, The Cochrane Collaboration, 2014) was used to conduct the meta-analysis. Heterogeneity was assessed by the Cochrane Q test and considered statistically significant if *p* ≤ 0.10. The *I*^2^ statistic was also used to quantify heterogeneity. Studies with an *I*^2^ statistic of 0–50% were recognized as having low heterogeneity [23]. To enhance the generalizability of our results, data between studies were pooled by using a random effects model. The weight of a study in a pooled analysis was evaluated by employing the inverse variance method. Data of similar measurement instruments were pooled and presented as the mean difference (MD). Deviating from the study protocol, the data of similar outcomes measured with different measurement instruments were also pooled and presented as the standardized mean difference (SMD). To evaluate the impact of individual research on the pooled results, a sensitivity analysis was performed by removing trials adopting MET program which were quite different from others, and recalculating the combined estimates on the remaining researches. All tests were considered significant at two-tailed *p* < 0.05.

## Results

### Search results

**Fig. 1:**
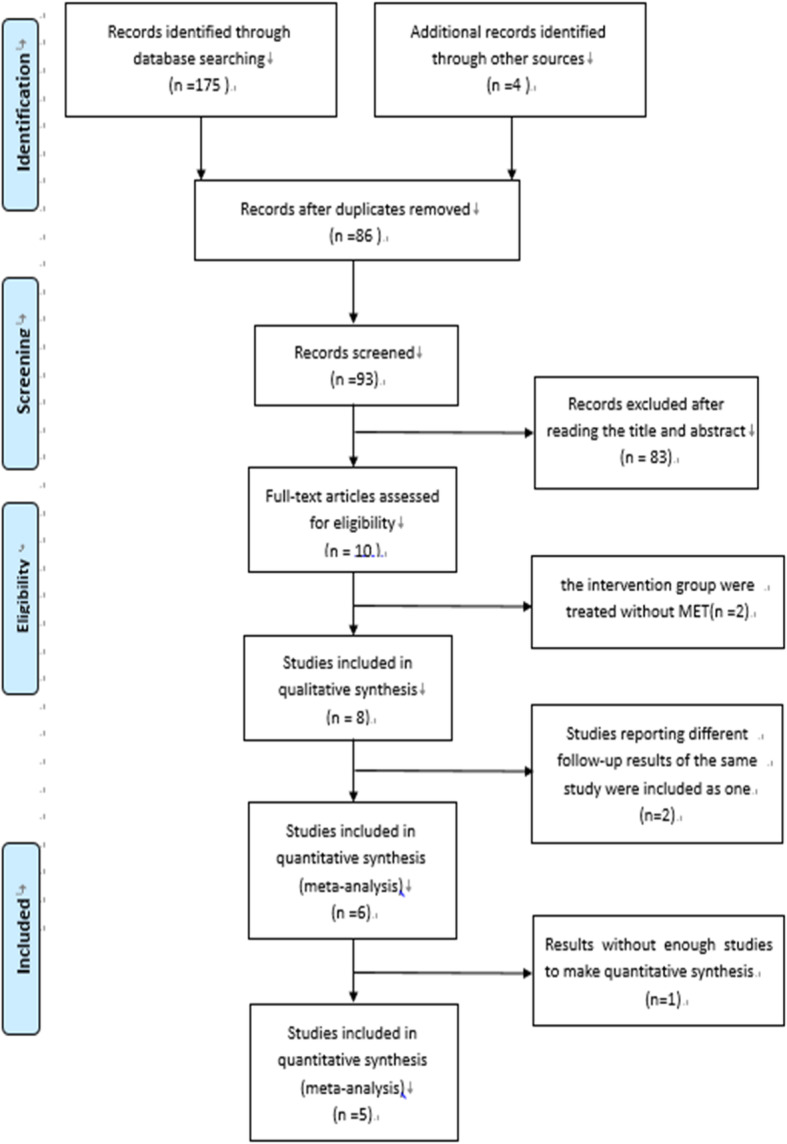
PRISMA flow diagram for the study selection process

The detailed retrieval process was shown in PRISMA flow diagram(Fig. 1). The search terms described above identified 175 references. A manual search of reference lists yielded 4 additional references. After removing duplicates, 93 studies were carefully assessed based on their title and abstract. Of the possibly eligible studies for inclusion, 2 were excluded because the intervention group only adopted APM but no MET [24, 25]. Thus, 8 studies were extracted [11, 12, 14, 15, 26–29]. In the case that two RCTs reported results of the same trial at different follow-up times, we considered them as one. Finally, the remaining 6 studies [11, 12, 14, 26, 27, 29] were included in this systematic review and meta-analysis. And we summarized the characteristics of the included trials [11, 12, 14, 15, 26–29] in Tables 1 and 2.

**Table 1:** Characteristics of the participants included in the review

Author, year	Participants		Gender (M/F)		Mean age (year)		Degenerative grade
	APM + MET	MET	APM + MET	MET	APM + MET	MET	
Gauffin 2014	75	75	53/22	56/19	54 ± 5	54 ± 6	Ahlback 0
Herrilin 2007	47	43	28/19	27/16	54 ± 5	56 ± 5	Ahlbacks 0 or 1
Herrilin 2013	47	49	28/19	30/19	54 ± 5	56 ± 6	Ahlbacks 0 or 1
Katz 2013	161	169	71/90	72/97	59 ± 8	58 ± 7	Kellgren-Lawrence grades 0 to 3
Yim 2013	50	52	9/41	12/40	55 ± 10	58 ± 11	Kellgren-Lawrence 0 or 1
Sihvonen 2014	70	76	42/28	47/29	52 ± 7	52 ± 7	Kellgrene-Lawrence grade 0 or1
Stensrud 2015	42	40	26/16	27/13	48.6 ± 6.4	49.2 ± 6.4	Kellgren-Lawrence 0 or 1
Sihvonen 2018	70	76	42/28	47/29	52 ± 7	52 ± 7	Kellgrene-Lawrence grades 0 or 1

**Table 2:** Summary of intervention information for each study

Author, year	Follow-up time	Symptom duration	Modalities of exercise	MET program		
				Duration (week)	Frequency (*p* week)	Session duration (min)
Gauffin 2014	3, 12 months	more than 3 months	gym or home exercise	12	2	30–40
Herrilin 2007	8 weeks, 6 months	2–6 months	home exercise	8	2	40–60
Herrilin 2013	24, 60 months	2–6 months	home exercise	8	2	40–60
Katz 2013	6, 12 months	more than 1 month	progressive home exercise	6	2	Not given
Yim 2013	24 months	8.2 months	home exercise	11	3	60
Sihvonen 2014	2, 6, 12 months	more than 3 months	graduated home-based exercise	Not given	5	10–15
Stensrud 2015	3 months	7.5 months	gym exercise	12 weeks	2–3	60–80
Sihvonen 2018	24 months	more than 3 months	graduated home-based exercise	Not given	5	10–15

### Study characteristics

The sample size of the extracted studies ranged from 93 to 351. All included RCTs were published between 2007 and 2017. Overall, 445 participants were randomly allocated to APM followed by exercise group, and 434 participants were in the exercise group. The average age ranged from 48 to 59 years and females accounted for 47.8% of all enrolled participants. The follow-up time was 2 to 60 months. In terms of evaluation of knee osteoarthritis grade, two studies used Ahlbacks classification [11, 27], and four studies adopted Kellgren-Lawrence classification [12, 14, 26, 29]. With respect to the MET program, the duration was ranging from 6 weeks to 3 months, and the frequency was ranging from twice to five times a week. The details of each study were summarized in Tables 1 and 2.

### Meta-analysis

**Fig. 2:**
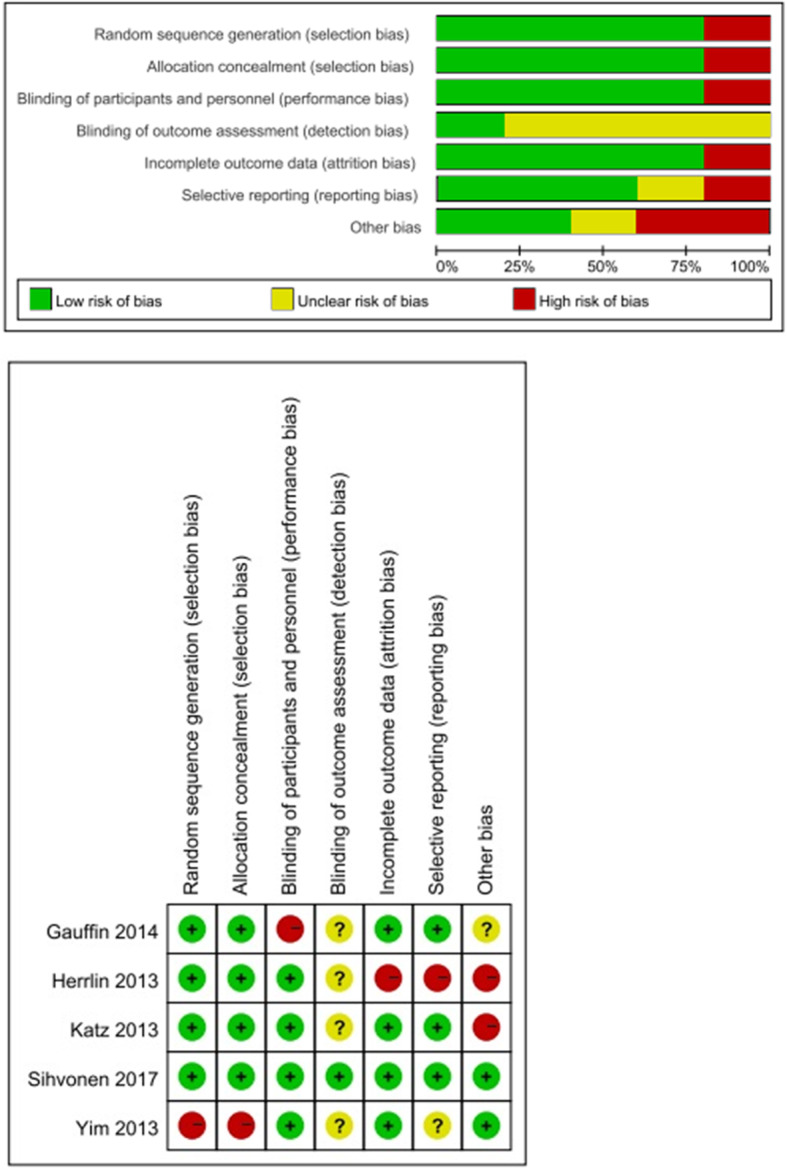
Risk of bias assessment

Assessments of the outcomes were conducted at 2 to 3, 6, 12, and 24 months, respectively. Clinical results were reported as follows: pain relief of knee, physical function, and activity level. We only shown the significant results in the text. An overview of the estimated risk of bias for each study was presented in Fig. 2.

#### Pain relief

**Fig. 3:**
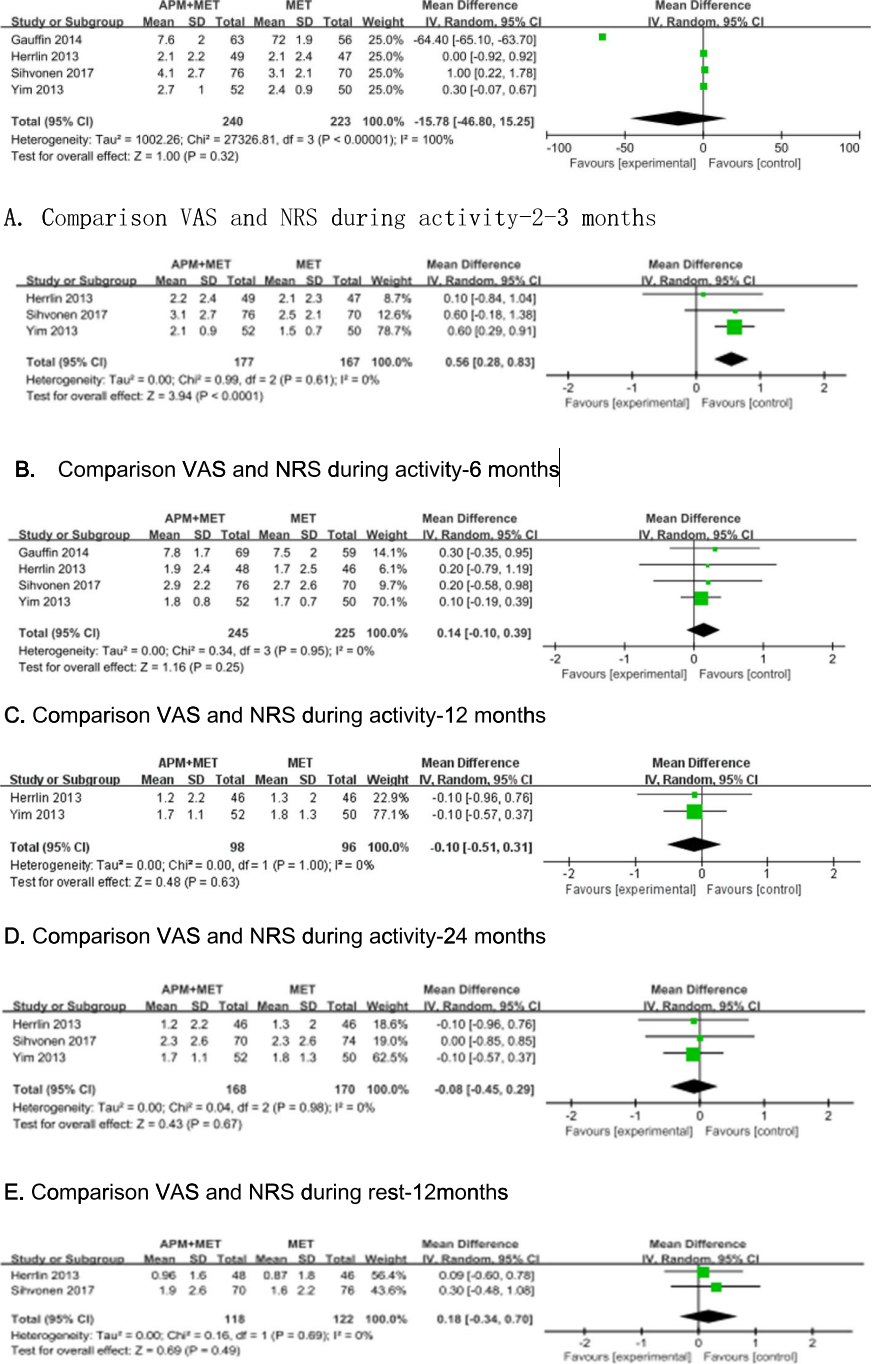
Forest plots showing the pain score measured by VAS and NRS at 2 to 3, 6, 12, and 24 months. **a** Comparison VAS and NRS during activity—2–3 months. **b** Comparison VAS and NRS during activity—6 months. **c** Comparison VAS and NRS during activity—12 months. **d** Comparison VAS and NRS during activity—24 months. **e** Comparison VAS and NRS during rest—12 months

**Fig. 4:**
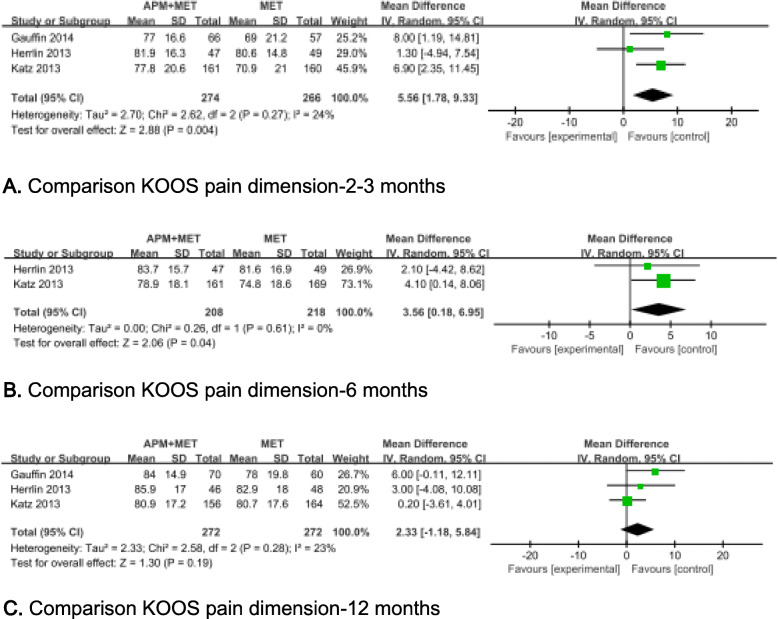
Forest plots showing the pain score measured by KOOS pain dimension at 2 to 3, 6, and 12 months. **a** Comparison KOOS pain dimension—2–3 months. **b** Comparison KOOS pain dimension—6 months. **c** Comparison KOOS pain dimension—12 months

Four studies used the visual analogue scale (VAS) and numerical rating scale (NRS) [11, 14, 26, 27], and three studies employed the Knee injury and Osteoarthritis Outcome Score (KOOS) Pain dimension [11, 12, 27] to assess the effects of treatments on pain control. Data from four studies [11, 14, 27] including 470 patients reported VAS or NRS score of target knee after a period ranging from 8 weeks to 60 months. It shown that there were statistically significant differences between two groups during activity only at 6 months after intervention (MD = 0.56, 95%CI 0.28 to 0.83, *p* < 0.0001; Chi^2^ = 0.99, df = 2, *P* = 0.61; *I*^2^ = 0%, Fig. 3b). Three studies [12, 26–28] including 549 patients mentioned KOOS Pain dimension. Significant differences were found between the two groups both at 2 to 3 months (MD = 5.58, 95%CI 1.82 to 9.34, *p* = 0.004; Chi^2^ = 2.64, df = 2, *P* = 0.27; *I*^2^ = 24%, Fig. 4a) and at 6 months (MD = 3.56, 95%CI 0.18 to 6.95, *p* = 0.04; Chi^2^ = 0.26, df = 1, *P* = 0.61; *I*^2^ = 0%, Fig. 4b) after the planned treatments were undertaken.

#### Physical function

**Fig. 5:**
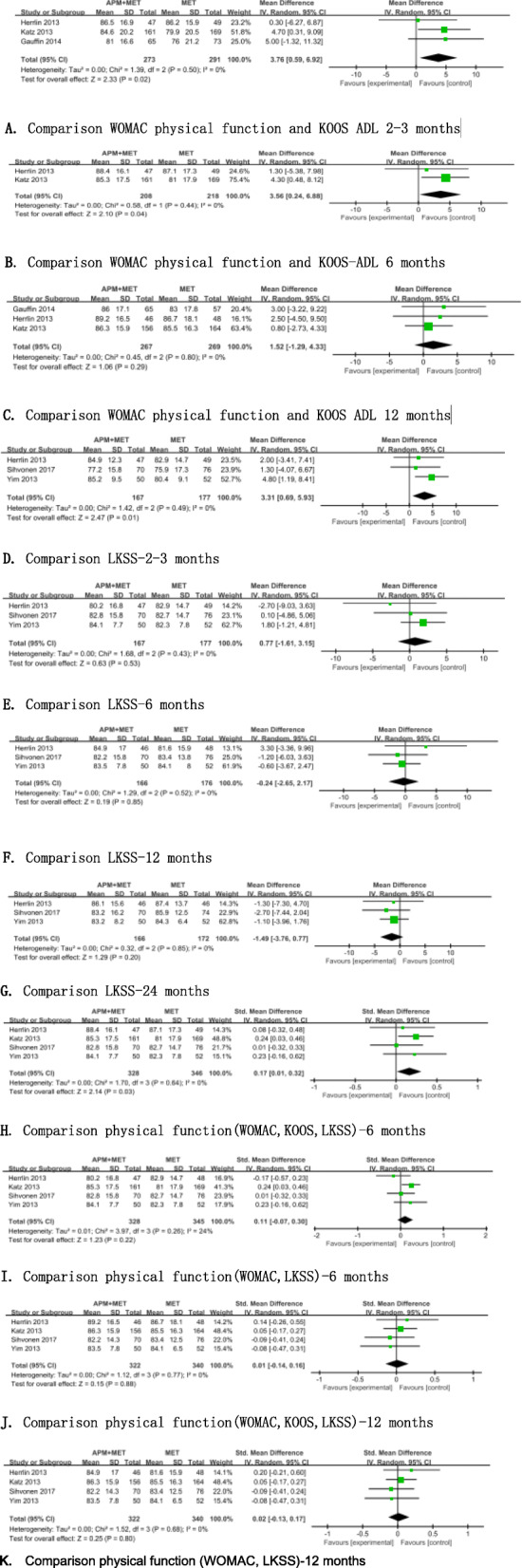
Forest plots showing the physical function measured by WOMAC, KOSS and LKSS at 2 to 3, 6, and 12 months. **a** Comparison WOMAC physical function and KOOS ADL 2–3 months. **b** Comparison WOMAC physical function and KOOS-ADL 6 months. **c** Comparison WOMAC physical function and KOOS ADL 12 months. **d** Comparison LKSS 2–3 months. **e** Comparison LKSS 6 months. **f** Comparison LKSS 12 months. **g** Comparison LKSS-24 months. **h** Comparison physical function(WOMAC, KOOS, LKSS) 6 months. **i** Comparison physical function (WOMAC, LKSS) 6 months. **j** Comparison physical function (WOMAC, KOOS, LKSS) 12 months. **k** Comparison physical function (WOMAC, LKSS) 12 months

To evaluate the physical function, different methods were employed in the included articles: Western Ontario and McMaster Universities Osteoarthritis Index (WOMAC), KOOS, and the Lysholm Knee Scoring Scale (LKSS). The same items were used in the WOMAC physical function dimension and the KOOS function in daily living dimension of activities of daily living, so they could be pooled directly. Finally, three trials including 549 patients were extracted [12, 27, 28] (Fig. 5). When these results were pooled, significantly favorable outcomes in the APM followed by exercise group at 2 to 3 months (MD 3.76, 95% CI 0.59 to 6.92, *p* = 0.02; Chi^2^ = 1.39, df = 2 (*P* = 0.50); *I*^2^ = 0%, Fig. 5a) and at 6 months (MD 3.56, 95% CI 0.24 to 6.88, *p* = 0.01; Chi^2^ = 0.58, df = 1 (*P* = 0.44); *I*^2^ = 0%, Fig. 5b) were found. Data from three studies [11, 14, 26] enrolling 349 patients adopted LKSS. Statistically significant differences were observed between the two groups only at 2 to 3 months after the therapy (MD 3.31, 95% CI 0.69 to 5.93, *p* = 0.01; Chi^2^ = 1.42, df = 2, *P* = 0.49; *I*^2^ = 0% Fig. 5d). Statistically significant differences were observed between groups only at the follow-up time of 6 months when pooling the different measurements (i.e., WOMAC, KOOS, and LKSS) together (MD 0.17, 95% CI 0.01 to 0.32, *p* = 0.03; Chi^2^ = 1.70, df = 3 (*P* = 0.64); *I*^2^ = 0%, Fig. 5h)

#### Activity level

**Fig. 6:**
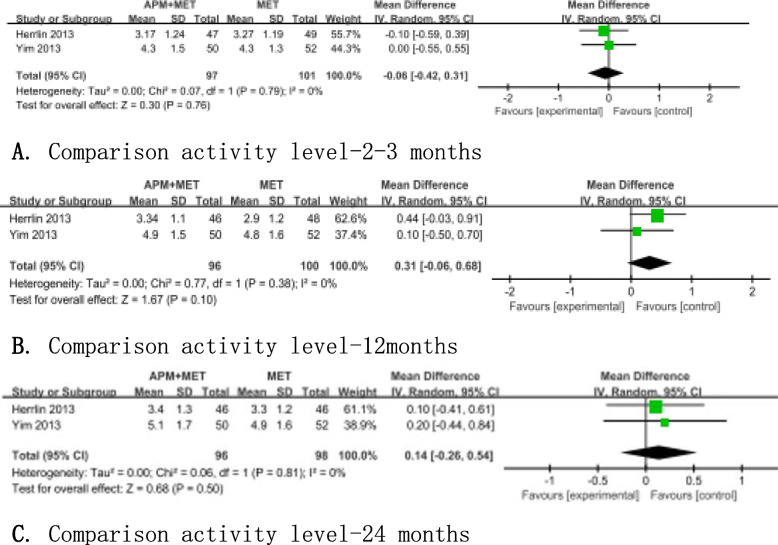
Forest plots showing the activity level at 2 to 3 months, 12 months, and 24 months. **a** Comparison activity level 2–3 months. **b** Comparison activity level 12 months. **c** Comparison activity level 24 months

Tegner Activity Scale score was used in two articles [11, 14] enrolling 198 patients to evaluate the activity level. No significant differences between two groups were found at the follow-up time of 2 to 3 months, 12 months, and 24 months (Fig. 6).

#### Sensitivity analyses

Additional sensitivity analyses were performed to confirm the results. We eliminated a study applying MET program (five times a week) quite different from others [26]. And the effect sizes did not change significantly (shown in Additional file 3).

## Discussion

We found that APM followed by structured MET was more effective compared to MET alone in terms of VAS score, KOOS Pain dimension, Lysholm Knee Scoring Scale, WOMAC, and KOSS function score within 6 months. These findings revealed that APM followed by MET may improve both pain control and physical function in patients with degenerative meniscal tear in a short-term. However, it was found that MET could be as effective as APM + MET both in the 12th and 24th months’ follow-up since the completion of interventions.

There was low statistical heterogeneity of the main positive outcomes. To increase the strength of the conclusions, we pooled the data from the included studies despite the usage of outcome measures that were various in the extracted articles. Most of the results were consistent, which improved the quality of evidence. In addition, we conducted the sensitivity analysis, and it also confirmed the results. Totally, the efficacy of APM with MET in patients with a meniscal tear was explored in 5 recent RCTs. When comparing the included studies, four of them did not show statistically or clinically significant differences in symptomatic outcomes between participants in the group of APM with MET or MET alone [11, 12, 14, 26]. One study demonstrated statistically significant and clinically significant strengths for surgery [27]. The only one [27] which shown significant improvements compared with exercises differed from other studies might because of the higher participation rate and lower rate of crossover. Therefore, it may be important to figure out the reasons of crossover in order to guarantee the results more credible for the future researches. The factors that some studies [30–32] identified points toward combination of characteristics putting participants at risk for crossover: intolerable pain and relatively short duration symptoms. Future studies may need to strive to keep these participants in non-operative group.

Recently, meta-analyses were published to compare APM with conservative treatment for non-obstructive meniscal tears [20]. It indicated that statistically beneficial outcomes of APM were found up to 6 months for physical function (LKSS at 2 to 3 months, WOMAC and KOOS at 6 months, and LKSS, KOOS, and WOMAC at 6 months) and pain relief (KOOS and VAS at 6 months), and there were no differences at longer follow-up. Obviously, these results were similar with our main outcomes. However, whether these differences were of clinical influences was unconvinced, because only small significant differences supported surgery followed MET within 6 months but not at longer follow-up. A trial indicated that APM + MET was not better than MET alone in terms of both radiographic evidence and patient-reported results after 5 years [11]. More studies should focus more on the differences of long-term clinical outcomes between APM + MET and MET.

Besides, other aspects should also be taken into considerations. Østeras et a. l[24] described that the MET group (3 months,3 times per week) has less depression and anxiety compared with APM group at the end of treatment 3 months. In addition, supervised exercise therapy program (12 weeks, 24–36 sessions) might result in greater improvements in isokinetic quadriceps strength compared with APM + MET at the 3-month follow-up time [29]. General health measured with the Short Form 36 was reported in one trial, and there were no significant differences between groups after 6 and 12 months [12]. As for the patients’ satisfaction, two groups shown similar data [14]. And no significant between-group differences in the incidence of specific or overall adverse events were found [12]. There were no doubts that the economic cost of APM + MET was higher than MET alone. And such information could add very valuable reference, particularly to both the health care providers and the patients when making the clinical treatment decision. Besides, the prevalent occurrence of osteoarthritis after APM remains an issue [33, 34]. To our knowledge, there might be a large gap between clinical reality and the conclusions of present articles preferring APM as the prior choice of treatment for degenerative meniscus tears. Choosing the proper treatment could be challenging because multiple factors should be taken into considerations, and APM may only be considered when the response to non-surgical treatment has not been satisfactory, and after comprehensively clinical and radiological assessments [35, 36]. Besides, the British Journal of Medicine recently published a clinical practice guideline that even suggested “using number of arthroscopies per-formed in patients with degenerative knee disease as an indicator of quality care” [37]. Therefore, the cost performance when combining APM with MET for treating degenerative meniscus tears should be carefully interpreted. And the potential of MET to treat DMT should be valued.

**Our systematic review had the following limitations**: (1) Only five trials evaluating a total of 797 subjects were included in this meta-analysis; if more researches available, the statistical efficacy of analysis might enhance. (2) Most of included trials were difficult to interpret because between 20% and 30% of participants initially assigned to the non-operative group crossed over to perform APM. (3) The follow-up duration was not long enough in some included studies. Long-term follow-up trials should be performed in the future. (4) Among-study heterogeneity was unavoidable because of the use of different grades of degenerative meniscal tear of patients and various programs of exercises. Despite several limitations existed, we carefully retrieved available studies based on strict inclusion criteria to ensure high quality. Moreover, Cochrane Handbook and PRISMA guidelines were employed to determine the quality of results contained in the extracted articles.

## Conclusions

No systematic review and meta-analysis were found that evaluated the effectiveness of MET alone compared to APM followed by MET in middle-aged patients with DMT. Despite the small number of patients and the heterogeneity in the extracted RCTs in this study, favorable results of APM + MET at short-term were found for pain control and physical function compared to MET, but there were no differences at longer follow-up. Our results supported the potential of MET that could be comparable to APM + MET regarding to the improvement of pain control and physical function in patients with DMT. Therefore, the clinical applicability of APM + MET compared with MET should be interpreted carefully, and the potential of MET to treat DMT should be valued.

## Supplementary information

**Additional file 1.** PRISMA 2009 Checklist.

**Additional file 2.** Search strategy on PubMed.

**Additional file 3.** Sensitivity analysis by excluding the study of Sihvonen et al.

## Data Availability

The authors declare that all the data supporting the findings of this study are available within the article and its supplementary information files.

## Abbreviations

APM: Arthroscopic partial meniscectom
DMT: Degenerative meniscal tear
KOOS: Knee injury and Osteoarthritis Outcome Score
LKSS: Lysholm Knee Scoring Scale
MD: Mean difference
MET: Medical exercise therapy
NRS: Numerical rating scale
RCTs: Randomized controlled trials
SMD: Standardized mean difference
VAS: Visual analog scale
WOMAC: Western Ontario and McMaster Universities Osteoarthritis Index
