# Supplementary material for: Medical exercise therapy alone versus arthroscopic partial meniscectomy followed by medical exercise therapy for degenerative meniscal tear: a systematic review and meta-analysis of randomized controlled trials
Source: J Orthop Surg Res. 2020 Jun 15;15:219. doi: 10.1186/s13018-020-01741-3 (PMC7296921; doi:10.1186/s13018-020-01741-3)
Supplement: Supplementary file 2 — Additional file 2. Search strategy on PubMed. [file 13018_2020_1741_MOESM2_ESM.docx]

Supplemental material 1: Search strategy on PubMed

#1 degenerative meniscus

#2 degenerative meniscal tear

#3 degenerative meniscal lesion

#4 Search (#1 OR #2 OR #3)

#5 "exercise"[Mesh]

#6 physical treatment

#7 non-operative treatment

#8 [conservative](javascript:;) [treatment](javascript:;)

#9 Search (#5 OR #6 OR #7 OR #8)

#10 arthroscopic partial meniscectomy

#11 APM

#12 Search #10 OR #11

#13 Search (#4 AND # 9 AND # 12)

((((APM) OR arthroscopic partial meniscectomy)) AND (((("Exercise"[Mesh]) OR physical treatment) OR non-operative treatment) OR conservative treatment)) AND (((degenerative meniscus) OR degenerative meniscal tear) OR degenerative meniscal lesion)
