# Supplementary material for: Medical exercise therapy alone versus arthroscopic partial meniscectomy followed by medical exercise therapy for degenerative meniscal tear: a systematic review and meta-analysis of randomized controlled trials
Source: J Orthop Surg Res. 2020 Jun 15;15:219. doi: 10.1186/s13018-020-01741-3 (PMC7296921; doi:10.1186/s13018-020-01741-3)
Supplement: Supplementary file 3 — Additional file 3. Sensitivity analysis by excluding the study of Sihvonen et al. [file 13018_2020_1741_MOESM3_ESM.docx]

| Sensitivity analysis by excluding the study of Sihvonen et al | | | | |
| --- | --- | --- | --- | --- |
| Item | Remaining studies | P | MD/SMD  （95%CI） | Heterogeneity |
| Comparison VAS and NRS during activity-2-3 months | Herrlin2013  Yim2013  Gauffin2014 | 0.32 | -15.78  （-46.8,15.25） | I^2^=100%  P<0.00001 |
| Comparison VAS and NRS during activity-6 months | Herrlin2013  Yim2013 | 0.0003 | 0.55  （0.25-0.85） | I2=0%  P=0.32 |
| Comparison VAS and NRS during activity-12 months | Herrlin2013  Yim2013  Gauffin2014 | 0.29 | 0.14  （-0.12,0.39） | I2=0%  P=0.85 |
| Comparison LKSS-2-3 months | Herrlin2013  Yim2013 | 0.01 | 3.94  （0.93,6.94） | I^2^=0%  P=0.40 |
| Comparison LKSS-6 months | Herrlin2013  Yim2013 | 0.83 | 0.45  （-3.6,4.49） | I^2^=37%  P=0.21 |
| Comparison LKSS-12 months | Herrlin2013  Yim2013 | 0.91 | 0.18  （-2.88,3.25） | I^2^=8%  P=0.91 |
| Comparison physical function(WOMAC,KOOS,LKSS)-6 months | Herrlin2013  Yim2013  Katz2013 | 0.02 | 0.21  （0.04-0.38） | I^2^=0%  P=0.77 |
| Comparison physical function(WOMAC,LKSS)-6 months | Herrlin2013  Yim2013  Katz2013 | 0.26 | 0.14  （-0.10-0.38） | I^2^=38%  P=0.20 |
| Comparison physical function(WOMAC,KOOS,LKSS)-12 months | Herrlin2013  Yim2013  Katz2013 | 0.65 | 0.04  （-0.13-0.21） | I^2^=0%  P=0.73 |
| Comparison physical function(WOMAC,LKSS)-12 months | Herrlin2013  Yim2013  Katz2013 | 0.57 | 0.05  （-0.12-0.22） | I^2^=0%  P=0.62 |
